# Exosomes, Their Biogenesis and Role in Inter-Cellular Communication, Tumor Microenvironment and Cancer Immunotherapy

**DOI:** 10.3390/vaccines6040069

**Published:** 2018-09-26

**Authors:** Kishore Kumar Jella, Tahseen H. Nasti, Zhentian Li, Sudarshan R. Malla, Zachary S. Buchwald, Mohammad K Khan

**Affiliations:** 1 Department of Radiation Oncology, Emory University, Atlanta, GA 30322, USA; zhentian.li@emory.edu (Z.L.); zachary.scott.buchwald@emory.edu (Z.S.B.); 2 Department of Microbiology and Immunology, Emory University, Atlanta, GA 30322, USA; tah.nasti@emory.edu; 3 Division of Renal Medicine, Emory University, Atlanta, GA 30322, USA; sudarshan.malla@emory.edu

**Keywords:** exosomes, biogenesis, exosome communication, tumor microenvironment, immunotherapy

## Abstract

Exosomes are extracellular vesicles ranging from 30 to 150 nm in diameter that contain molecular constituents of their host cells. They are released from different types of cells ranging from immune to tumor cells and play an important role in intercellular communication. Exosomes can be manipulated by altering their host cells and can be loaded with products of interest such as specific drugs, proteins, DNA and RNA species. Due to their small size and the unique composition of their lipid bilayer, exosomes are capable of reaching different cell types where they alter the pathophysiological conditions of the recipient cells. There is growing evidence that exosomes are used as vehicles that can modulate the immune system and play an important role in cancer progression. The cross communication between the tumors and the cells of the immune system has gained attention in various immunotherapeutic approaches for several cancer types. In this review, we discuss the exosome biogenesis, their role in inter-cellular communication, and their capacity to modulate the immune system as a part of future cancer immunotherapeutic approaches and their potential to serve as biomarkers of therapy response.

## 1. Introduction

Exosomes are double-layered vesicles ranging from 30−150 nm [1,2,3] in diameter with a buoyant density ranging from 1.13−1.19 g·mL^−1^. The lipid bilayer of exosomes matches the cells that release them; the membrane layer of exosomes resembles with lipid profile such as cholesterol, sphingolipid, and phospholipid content of the parental cell of origin [4]. The double-layered lipid membrane loaded with peripheral and integral membrane proteins allows intercellular communication, and regulates various signaling pathways that are crucial for both inter and intra-cellular communication [5]. They were first discovered in sheep reticulocytes, and later observed in almost all mammalian cells analyzed, driving certain physiological responses in recipient cells [6]. Exosomes are formed by the inward budding of the membrane of specific late endosomes to form multivesicular bodies (MVBs). These MVBs fuse with plasma membranes to release their content outside the membrane [7]. Initially, exosomes were thought to function in cell waste management. However, it is now recognized that exosomes are key components for cell-to-cell communication. During the last decade of the 20th century, exosomes were identified to play a role in the presentation of B lymphocyte antigens, and later recognized to play a role in immune related functions [8]. During the early 21st century, several researchers reported the presence of various RNA species such as miRNA and mRNA within the exosomes [3,4,9]. These were found to serve as messengers for intercellular communication, and indicated to have a profound effect on target cells. Exosomes have also been reported to be active during various physiophathological conditions such as tumor suppression/progression, tumor immunity, and inflammation [10]. In this review, we discuss the role of exosomes, their biogenesis, their role in tumor development and their applications in cancer immunotherapy.

**Figure 1:**
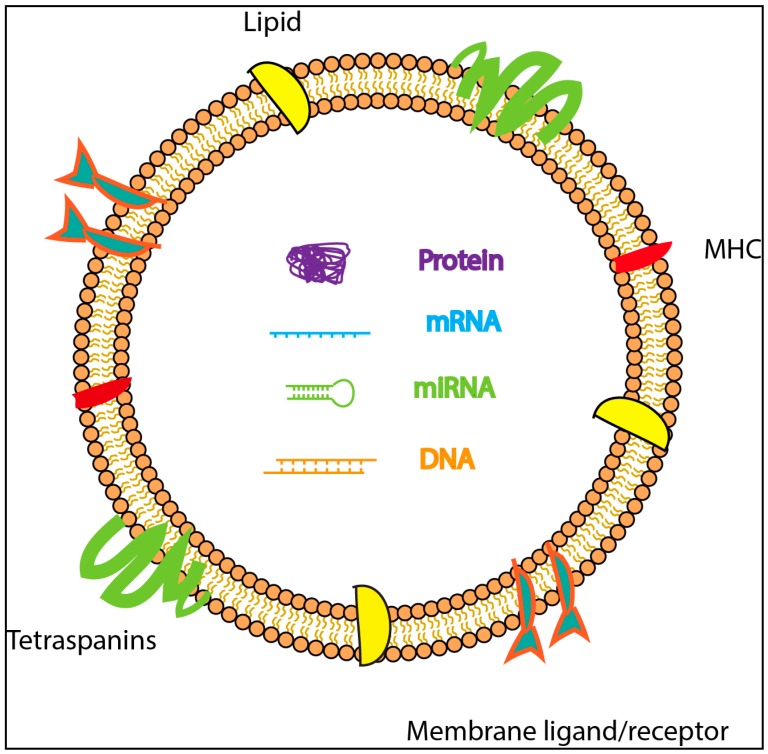
Schematic representation of exosome structure containing DNA, proteins and RNA species surrounded by a lipid bilayer with membrane ligands/receptors, tetraspanins and major histocompatibility complex (MHC).

The basic structure of exosome is described in Figure 1. According to Exocarta database (Version 5; http://exocarta.org), till date, 41,860 proteins, 3408 mRNAs and 2838 miRNAs have been reported to be present in exosomes derived from different cell types. The function of RNA present in the exosomes is not well understood; however, it is thought to transmit signals to distant sites, thus promoting and regulating the function of remote cells [11]. Exosomes circulating in the blood can interact with platelets and endothelial cells, in vivo [12]. They can also affect the development of disease processes such as cancer and autoimmune diseases [13,14].

### 1.1. Exosome Biogenesis

Circulating vesicles in blood are often composed of exosomes and microvesicles, and are difficult to isolate to maximum purity using current purification methods. Both exosomes and microvesicles originate from within the cell, but their biogenesis pathways are somewhat distinct. Microvesicles originate from the outward budding of the plasma membrane, whereas exosomes are formed by inward budding of outer membrane [15,16,17]. The inward budding of plasma membrane is the origination of early endosomes, which then, later mature into late endosomes. Some of these late endosomes then become MVBs. Isolation of pure exosomes vs pure microvesicle is difficult, and the current methods only allow for separation of small extracellular vesicles from large/medium extracellular vesicles [5]. In contrast, microvesicles are simply formed by shedding of plasma membrane, or by exocytosis [18].

Collectively known as MVBs, they have two functions: (1) they can fuse with lysosomes and become involved in degradation pathway or, (2) fuse with the cell membrane to release smaller vesicles into the extracellular environment [16]. Late endosomes contain many small vesicles. The smaller vesicles that are contained within the cells are referred to as intraluminal endosomal vesicles [16]. The small vesicles that are released to the extra-cellular environment are termed as exosomes (Figure 1), containing many of the cellular contents from which they are formed. Though the exact mechanism of biogenesis of exosomes is not fully understood, several recent reports suggest that the syndecan heparin sulphate proteoglycans and syntenin regulate the formation of exosomes [19,20]. The release of exosomes is regulated by Rab GTPase pathway [21,22]. The delivery and transfer of exosomes to their recipient cells is regulated by “Endosomal Sorting Complexes Required for Transport” (ESCRTs), Ca^+2^ channels and cellular pH levels [23,24,25,26,27,28]. Silencing of ALIX proteins also regulates the release of exosomes [29]. The mechanism of cargo sorting is not fully understood, but it was reported that the ESCRT-dependent endo-lysosomal pathway is important during exosomes biogenesis and cargo sorting processes and, also involves the syndecan–syntenin–ALIX axis [20,21]. The released exosomes are taken up by the recipient cells through receptor-mediated endocytosis or by the receptor-ligand fusion process [30].

### 1.2. Exosomal Mediated Communication Between Cells

Several studies confirm that exosomes interact and communicate with recipient cells [9]. The exact mechanism of their interaction with cells is mainly based on in vitro studies. Some mechanisms that have been proposed are: (1) exosomes bind to the surface of a recipient cell through adhesion molecules on exosomes; (2) fusion of vesicle after adhesion with plasma membrane; (3) receptor mediated endocytosis, and (4) phagocytosis by internalizing the vesicles into endocytic compartments. These interactions between target cells and exosomes can lead to transfer of membrane receptors, growth factors bound on the surface of exosomes, delivery of specific proteins to target cells, and transfer of genetic material [31].

**Figure 2:**
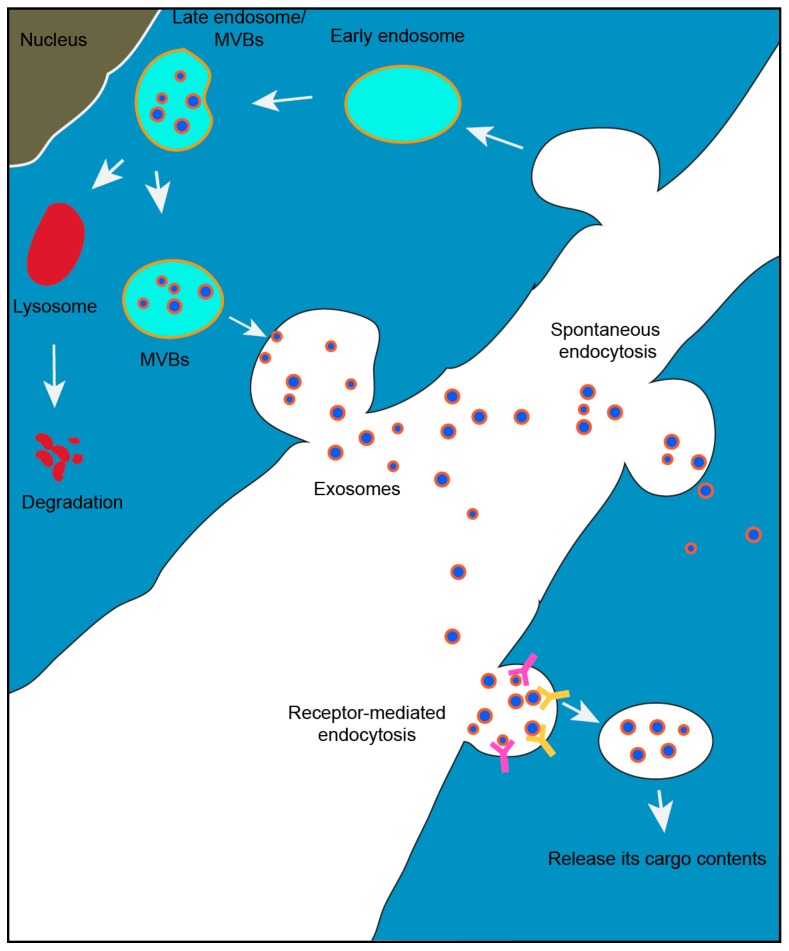
Exosome biogenesis begins with budding into early endosome and further matures into late endosome, collectively known as multivesicular bodies (MVBs). Thus, formed multivesicular bodies fuse with plasma membrane to release exosomes into outer cellular environment to communicate with other cells. The late endosomes fuse with the lysosome to undergo degradation pathway. In recipient cells, exosomes are taken up by receptor-mediated endocytosis process and release their cargo contents.

**Figure 3:**
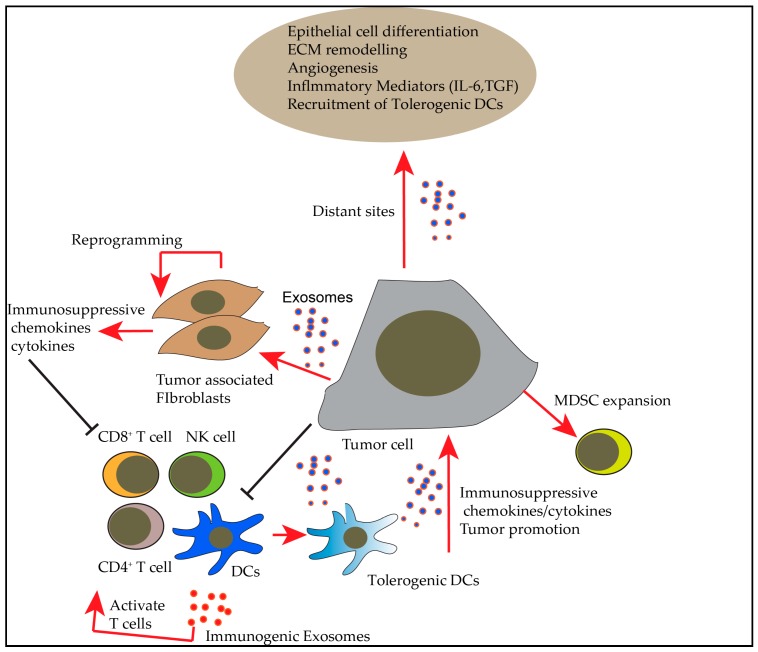
Summary of effects of exosomes on different immune cells and ways by which they promote tumor development and metastasis.

A well-known mechanism of intercellular communication is through signaling molecules such as proteins, which interact with the receptors present on the surface of the target cells. Exosomes can transfer wide range of molecules such as proteins, RNA, DNA and lipids, which regulate various pathways in recipient cells present at particular sites. Existence of both miRNA and mRNA within exosomes, and the shuttling of these exosomes to recipient cells are believed to take part in cell to cell communication [31]. Identification of DNA and RNA in exosomes that were isolated by ultracentrifugation approach, but not confirmed by further purification steps such as differential centrifugation needs further analysis [32,33]. Many studies revealed the presence of different non-coding RNA species along with cellular RNA within exosomes targeted to the cell of interest [34]. The most enriched RNA within exosomes includes mRNA that shuttles via exosomes, small non-coding RNA (Y-RNA), miRNA and transfer RNA (tRNA). Shuttling of these conserved short RNA species is associated with the function of various genes involved in cellular regulation [34]. The amount of miRNA released per exosome is different for different cells and tissues, and can range from low to very high. For example, the amount is very low within the plasma, serum, seminal fluid, cerebrospinal fluid and even some cells such as mast cells, dendritic cells and ovarian tumor cells [35]. The amount of miRNA obtained from these exosomes is very low; however, they are highly specific towards their targets and carry specific functions [36]. Communications between the donor and recipient cells are presented in Figure 2 and Figure 3. Figure 2 represents the formation and release of exosomes to the extra-cellular environment, and Figure 3 represents the various interactions that the released exosomes could cause within a host.

### 1.3. Isolation and Characterization of Exosomes

The basic technique that has been widely used to purify exosomes is the ultracentrifugation method; however, achieving maximum purity of the exosomes is a major hurdle due to contaminating materials such as microvesicles, apoptotic debris or other impurities [4,37,38]. Although, ultracentrifugation method is considered a gold standard, other methods used include density gradient purification, affinity based purification and the precipitation method [39]. The basic purification steps are performed by centrifugation at 230× *g* to remove cellular debris. Microvesicles are removed at 10,000× *g* and exosomes are pelleted and washed at 100,000× *g* [40,41]. Ultracentrifugation is laborious and time-consuming but is effective in removing contaminants such as ribosomes or other protein complexes [42,43]. In the density gradient purification method, exosomes are collected based on buoyant density using discontinuous gradients of Opti-prep media or sucrose solution. The major disadvantage of this technique is the loss of sample during the purification process.

Several manufacturers (Invitrogen, Qiagen, etc.) offer kits designed for isolation of exosomes by precipitation methods. The advantage is that exosomes can be isolated by low speed centrifugation process. The limitations include tedious downstream processing due to use of various columns for further purification [44]. Exosomes isolated by affinity capture methods use the targeting antigens (CD9, CD81 and Flotilin-1) present on the surface of exosomes for binding and purification. The affinity purification procedure has been described in detail elsewhere [43,45,46]. The affinity based purification method reduces the contamination of other materials and allows isolation of pure exosome populations [46,47,48]. Table 1 presents the advantages and disadvantages of various exosome purification methodologies.

The isolated exosomes can be characterized by Western blotting that identifies exosomal markers such as TSG101, ALIX, Flotillin-1 and tetraspanins such as CD9, CD81, and CD63. Transmission electron microscopy helps to identify the double-layered lipid membrane and their diameter. NanoSight method measures the size and distribution of exosomes. Dynamic light scattering instruments measure the size of exosomes and microvesicles by measuring the fluctuations of laser light particles passing through the suspension of extracellular vesicles [49]. Tunable resistive pulse sensing instrument performs the direct measurement of size and distribution of exosomes using the qNano system [50].

## 2. Exosomes in Tumor Microenvironment and their Role in Immunosuppression

Exosomes are important components and regulators of the tumor microenvironment. It has been shown that tumor cells have a higher propensity to secrete larger quantity of exosomes [59]. The contents of tumor exosomes include protein and RNA species, whose quality and diversity are different to normal cell derived exosomes [60,61]. Involvement of exosomes in promoting tumor progression has been investigated [62]. Studies have shown that tumor exosomes are involved in communication between tumor and normal cells, and help promote tumor growth and invasion through MAPK/ERK signaling and miR-338/MACC1/MET pathways [63,64], leading to changes that assist tumor progression. Exosomes released by tumor cells have also been found to educate adipocytes by creating a suitable environment suitable for tumors, and help promote tumor progression [65]. A proteomic study of colorectal cancer cells harboring *Kras* mutation showed an enrichment of KRAS, EGFR, and SRC family kinases in exosomes. These exosomes enhanced the invasiveness of recipient cells, an implication of non-cell autonomous effects of mutant *Kras* mediated by exosomes [66]. Melo et al. reported that breast cancer associated exosomes contain pre-miRNAs, and the RNA-induced silencing complex (RISC) related proteins Dicer, AGO2 and TRBP, which are essential during miRNA biogenesis. This study demonstrated that the breast cancer cell secreted exosomes were able to transform normal cells on a Dicer dependent manner [67]. Another study showed that MDA-231 breast cancer derived exosomes were able to prime the hepatic niche, which facilitated the seeding of the cancer cells to the liver. Interestingly, the miRNA contents were significantly different in the tumor derived exosomes, which includes a distinct set of miRNAs involved in epithelial cell differentiation [68], and the exosomes from normal cells. A more recent study confirmed presence of mRNA as well as miRNA inside B16F0 tumor cells derived exosomes by microarray, to further examine the biological functions of exosomes. The authors treated cytotoxic T lymphocytes with B16F0 cells derived exosomes. They found 4 of the top 20 mRNAs expressed in B16F0 exosomes (*Wsb2*, *Fam168b*, *Cmtm4* and *Ptpn14*) were then upregulated in the recipient cells [69] after treatment with exosomes, indicating that selected mRNAs are capable of shuttling between donor and recipient cells through use of exosomes.

Tumor metastasis is a complex process, which requires the tumor cells to adapt and grow in a new microenvironment. Rossi et al. in 2018 reviewed that exosomes derived from bone cells can increase proliferation of cancer cells, and mediate communication between tumor and bone cells [70]. Exosomes are able to migrate to a distant location and involve in metastasis process [71]. Indeed, evidence showed that melanoma-derived exosomes were capable of promoting pre-metastatic niche formation by transferring oncogene MET (mesenchymal to epithelial transition) to recipient cells. This study also demonstrated that tumor exosomes exposure caused an upregulation of a distinct set of inflammatory and ECM-related genes [71]. Exosomal TGFβ was also found to be correlated with lymphatic metastasis in a gastric cancer study [72]. In addition to single exosomal protein factors, multiple miRNAs have also been identified in promoting tumor metastasis. Xu et al. reported that lung adenocarcinoma cell derived exosomal miR-21 facilitated osteoclastogenesis, which is correlated to tumor osteolytic metastasis [73]. Another study by Yang et al. demonstrated that exosomal miR-423-5p promote cancer cell growth and metastasis by repression of SUFU protein expression, which enhance the proliferation and migration in recipient gastric cells [74]. Gong et al. reported miR-675 from metastatic osteosarcoma promotes cell migration and invasion through regulation of CALN1 protein [75]. Exosomal miR-103 was shown to promote metastasis by directly targeting junction proteins VE-Cadherin, p120-catenin and zonula occludens [76]. Exosomal miR23a from nasopharyngeal carcinoma was shown to promote angiogenesis by repressing gene *Tsga10* [77]. Redox homeostasis in the tumor microenvironment is another factor that stimulates exosomes secretion from tumor cells. This study also demonstrated that exosomes release from tumor cells in a hypoxic microenvironment facilitated angiogenesis and metastasis [78]. Exosomal miR-135b was shown to enhance angiogenesis from hypoxic multiple myeloma cells via the HIF-I signaling pathway [79].

Taken together, the tumor microenvironment corresponds to the interaction between tumor cells and non-transformed surrounding tissue, mediated by cell-cell direct interaction or signaling molecules in the extracellular matrix (ECM), which varies over space and time. Tumor derived exosomes were capable of promoting a favorable microenvironment for tumor growth, allowing cancer cells to survive, proliferate and disseminate [80]. Cancer exosomes also have the ability to accelerate angiogenesis by providing necessary nutrients to the tumor microenvironment [81,82]. A recent study showed that exosomes from tumor-associated fibroblasts were able to “smuggle” essential nutrients to tumor cells, made the tumor cells less oxygen-based energy dependent, and led to metabolic reprograming which promoted tumor growth under nutrient stressed conditions [83].

Furthermore, exosomes can also condition the tumor microenvironment and interact with T cells via antigen-presenting cells (APCs) to alter the immune responses within the tumor microenvironment. External insult on the tumors with radiation results in change of quality and quantity of exosomes. For example, Diamond et al. showed that tumor derived exosomes from irradiated tumors contain dsDNA that induce stimulation of Interferon signaling in recipient cells [84]. However, there is sufficient evidence that tumor-derived exosomes are forerunners of immune suppression. They transport immunosuppressive molecules and factors that interfere with immune cells such as APCs and program them to secrete immunosuppressive cytokines. They can directly or indirectly influence the development, maturation, and antitumor properties of immune cells by carrying the cargo from tumors at a distant site to a site of antigen presentation or the hub of immune cells, thus promoting the pro-tumor agenda. A summary of different ways by which exosomes influence immune responses and promote tumor development is depicted in Figure 3.

Exosomes can mediate immunosuppression through a number of mechanisms. They can deliver tolerogenic signals to immune cells and can polarize immune cells such as DCs to tolerogenic DCs, they can inhibit CD8, CD4 and NK cell proliferation, induce apoptosis of CD8 T cells, suppress NK cell activity, polarize cancer associated fibroblasts and drive expansion of regulatory T cells and Myeloid derived suppressor cells (MDSCs). These mechanisms are reviewed in detail by Whiteside [85]. Furthermore, exosomes can target immune therapies by above mechanisms and by binding and sequestering tumor-reactive antibodies and thus radically decrease the anti-tumor effect [86]. The following section does not address the negative effects of exosomes, but we focused on how exosomes have been utilized in cancer immune therapies.

## 3. Exosomes in Cancer Immunotherapy

Cancer is one of the primary causes of death in many parts of the world, including the United States [87]. First-line treatments such as chemotherapy and radiation therapy show limited efficacy in certain malignancies, but may also lead to severe toxicity. Novel therapies, including immune checkpoint inhibitors, anti-CTLA4 and anti-PD1/PDL1, have revolutionized the way these cancers are treated. By re-invigorating the patients’ immune system, these potent therapies are capable of using the body’s own immune cells, particularly the CD8^+^ T-cells, to mount an effective anti-tumor response. Although the potential of these therapies is widely appreciated, there is significant room for improvement. Immune checkpoint therapies are frequently ineffective, and there is also a possibility for severe autoimmunity. Many tumors, due to genetic, biological and other factors make some patients less likely to respond to these therapies. To overcome some of these obstacles, innovative approaches are needed that are less toxic and provide more frequent and long-lasting responses. One such treatment is the use of nanoparticles and specifically exosomes.

In recent years, many new antigen and drug delivery systems such as liposomes, niosomes, and various metal-based nanoparticles have been developed that can directly or indirectly target tumors with precision [88]. The main goal of the cancer immunotherapy is to stimulate the immunosuppressed host to recognize and eradicate tumor cells. Nanoparticles can be manipulated to target dendritic cells (DCs) and macrophages, thereby stimulating the immune system via the delivery of adjuvants and antigens [89]. This provides the innate immune system access to a wide range of tumor antigens, efficient antigen presentation, co-stimulation and hence effective activation of CD4 and CD8 T cells. Although nanoparticles are widely used in many drug formulations, these nanoparticle-drug formulations face unique challenges from both clinical and translational perspectives. The lack of long-term safety and ineffective targeting of desired cell types are some of the issues challenging the nanoparticle-based formulations [90]. Despite decades of exhaustive investigation, nanoparticles for cancer immunotherapies face biological, technological, and study design related challenges in clinic settings. However, research into a specific type of nanoparticles, exosomes, is yielding exciting results.

A major advantage of exosomes is their size in the nano range (30–150 nm) and they are shed by both normal and abnormal cells, and hence can be obtained from the host’s own cells. Almost all cells types including T, B, tumor and dendritic cells secrete them [91]. This is valuable as the exosome cargo can be modified and the particle destination altered by modifying the cells that secrete them, making targeting easy and accurate. Due to specific exosome cargo and the variety of biological processes they modulate, these particles can be exploited as drug, antigen and gene delivery systems, and they may be of tremendous benefit in cancer immunotherapy. The major advantages of exosomes compared to other nanoparticles are their extended circulating half-life, the fundamental ability to target cells, biocompatibility, and minimum toxicity issues [92,93]. Thus, they appear to be an excellent choice for cancer immunotherapy.

There are many diseases where exosomes have demonstrated efficacy in pre-clinical studies. In Parkinson’s disease, exosomes were successfully employed to deliver catalase across the blood brain barrier (BBB), resulting in an improvement in disease state [94]. Similar methodology can be used to prepare exosomes that can effectively deliver drugs and immunotherapeutic agents across the BBB in primary CNS malignancies (Glioma) or metastatic patients. Exosomes modified by altering their cell surface were loaded with a variety of immunotherapeutic agents to target specific cell types. The overexpression by chronic myeloid leukemia (CML) cells of the IL-3 receptor (IL3-R) compared to normal immune cells was used for targeting [95]. Human embryonic kidney (HEK293T) cells were modified to express fusion protein Lamp2b, an exosomal protein, and cytokine interleukin 3 (IL-3). These exosomes were further loaded with imatinib or with breakpoint cluster region protein-Abelson murine leukemia viral oncogene homolog 1 (BCR-ABL) siRNA that inhibited CML cells both in vitro and in vivo. Similar approaches can be used in different tumor types, including melanoma that express epidermal growth factor receptor (EGFR). The melanoma cells can be targeted by exosomes isolated from cells that are made to express fusion proteins of VEGF and LAMP2b. Another approach is using DC derived-exosomes to stimulate the immune system. These exosomes have been used as functional vesicles that have MHC/peptide complexes at their surface. These are efficient formulations that support promoting T cell-dependent anti-tumor activity [96]. However, in this Phase I trial, the observed clinical regressions were T cell independent. Further analysis in mouse models revealed that natural killer (NK) cells were responsible for the anti-tumor activity. These exosomes stimulated IL-15Ra and NKG2D, an orphan receptor expressed by NK cells, inducing NK cell activation and proliferation. Extending this finding to human studies, the DC exosomes expressed functional IL-15Ra and NKG2D ligand, which permits proliferation, activation and IFN-γ secretion by NK cells. NK cell activity was observed in 50% of the patients and hence provided the link between exosomes and tumor regression.

Using DC derived exosomes, others have treated human breast adenocarcinoma cells (SK-BR-3) [97]. They used these tumor cells to stimulate CD3^+^ T-cells that had previously been exposed to SK-BR3 antigens. The T-cells cultured with tumor cells exposed to DC-exosomes showed a significantly higher percentage of IFN-γ positive when compared to the cells exposed to non-DC-Exo-treated tumor cells. These data demonstrate that the incorporation of DC-exosomes by the tumor cells increased their ability to activate T-cells. Exosomes that originated from peptide-pulsed DCs, induce immune response by antigen presentation to T-cells. This implies that DC-derived exosomes have MHC-peptide complexes and co-stimulatory molecules on their membrane, thus arming them with efficient antigen presentation potential [98]. Additionally, in another study, exosomes derived from mouse cell lines expressing tumor antigen human mucin 1 (hMUC1), prompted an effective immune response and anti-tumor activity against hMUC1-expressing tumor cell growth in vivo [99].

Anti-tumor responses were observed in mesothelioma bearing mice that were administered exosomes obtained from malignant mesothelioma cells [100]. The observed response was due to tumor-derived antigens found in mesothelioma cells. Tumor-derived exosomes that contain both MHC class I/II molecules, important molecules in immune activation, have been widely used with demonstrated efficacy [101]. In Lee et al. they established B16F1 murine melanoma cell line (B16F1-CIITA) by transduction of the *CIITA* (Class II transactivator) gene. Exosomes from this cell line contained elevated levels of MHC class II and melanoma antigen Tyrosinase related protein (TRP)2. Incubation of these exosomes with DCs induced the expression of MHC class II and CD86. In vitro assays showed enhanced proliferation and IL-2 secretion from splenocytes in co-culture experiments with DCs. Compared to B16F1 derived exosomes, B16F1-CIITA-exosomes induced increased mRNA levels of inflammatory cytokines such as TNF-α, IL-12 and chemokine receptor CCR7. Furthermore, B16F1-CIITA-exosomes significantly inhibited tumor development in a dose-dependent fashion. B16F1-CIITA-exosomes immunized mice exhibited higher levels of IgG2a antibodies, IFN-γ and TRP2-specific CD8^+^ T cells. These data suggest that compared to parental exosomes, the B16F1-CIITA-exosomes are more efficient in inducing anti-tumor immune responses, suggesting a significant role of MHC class II tumor exosomes in cancer therapy. Thus, similar to DCs, the DC-derived exosomes are enriched with receptors and molecules important for antigen presentation and T-cell activation. These include molecules such as CD40, CD80, CD86, MHC class I, II etc., facilitate activation of both the innate and adaptive immune responses, thus increasing the quality of anti-tumor response [102,103].

The promising results demonstrated both in vitro and in preclinical models using exosomes, while clinical trials have also revealed the potential of exosomes as immunotherapeutic agents. Targeting cancer stem cells is considered to be one of the highest potential approaches for cancer treatment. Metastatic melanoma patients in Phase I clinical trials were treated with exosomes obtained from monocytic DCs of the same patients. The exosomes were loaded with MAGE tumor antigens and MHC type I or type II depending on the MAGE peptide. Although conclusions about efficacy were not made, the efficacy of exosome administration was established [104]. Another Phase I trial in non-small cell lung cancer (NSCLC) patients showed immune activation upon exosome treatment and delay in disease progression [105]. A single arm phase II trial of advanced NSCLC patients demonstrated that IFN-γ plus class I and II loaded exosomes were capable of enhancing NK cell-mediated anti-tumor immunity. Half of the participants experienced progression free survival for more than 4 months with a median overall survival (OS) of 15 months [106]. In colorectal cancer, a Phase I clinical trial was done using ascite-derived exosomes combined with GM-CSF treatment. The therapy showed a positive response with induction of tumor-specific antitumor cytotoxic T lymphocyte response [107].

In conclusion, exosomes are biological vesicles with dimensions in nano range that can play an important role in cancer immunotherapy. Their ability to transfer their cargo including proteins and DNA/RNA to target cells is powerful and can be an efficient tool in immunotherapy. Depending on the cells they originate from, exosomes can be immunostimulating or immunosuppressive, and therefore can be effective tools for cancer immunotherapies or autoimmune diseases. Although exosomes are promising tools for immunotherapy of cancer, in order to translate the findings into the clinical settings, issues such as development of an optimal purification method, the choice of exosome donor cells, type of loading procedure, and scale-up need to be addressed. Furthermore, the cost and time required to purify exosomes for human use has to be reasonable to be used in the clinic. Therefore, there is a need for development of an optimal isolation technique that can produce large amounts of exosomes at low cost. For quick and efficient treatment, the technology to produce exosomes from any source can be developed and used in the future.

## 4. Exosomes and Future Perspective in Disease

Due to their capacity of selective cell targeting, the potential for exosomes in the antigen, drug delivery and immune therapies is immense. The purity of exosomes is still a challenge and even very insignificant quantities of impurities such as protein aggregates and other DNA/RNA species can alter their impact and the outcome. Further developments in drug/nucleic acid loading are also necessary for improved therapeutic benefits from exosomes. Exosomes have started to garner interest in the fields of cancer therapeutics, immunotherapy, cancer diagnostics, and biomarker development. There is a need for serological biomarkers that can be used as a predictive marker of cancer immune therapies. Other techniques such as PET/CT that can lead to pseudo-progression are expensive for patients and are not reliable for measuring immunological responses. Furthermore, tumor micro-environmental changes cannot be accessed in real time with these techniques. Recently, it has been shown that PD-L1 on circulating exosomes correlates with positive response and varies during the course of the treatment [108]. Hence, to access these real time changes in tumor microenvironment due to immune responses, tumor exosomes may be used as a reliable biomarker. Lastly, due to their passive targeting, small size and their composition, they may be preferred over even nanoparticles. However, there are limitations to their isolation/purification. There is an inadequate understanding of their influence on the immune system, which needs further dissection. Refined and detailed clinical and preclinical studies that can address these deficiencies will lead to new exosome-based approaches that will transform cancer therapies and allow for the development of more individualized predictive biomarkers.

## Figures and Tables

**Table 1:** Exosome purification methods.

Methodology	Advantages	Disadvantages	References
Ultracentrifugation	Bulk purification of exosomes is easy	Time consuming, contaminating proteins	[40,41,51]
Density gradient centrifugation	High purity exosome	Loss of exosomes, skillful technique	[7,52,53]
Ultrafiltration	Good exosome yield and quick isolation method	Purity is less	[54,55]
Immunoaffinity method	Exosome enrichment based on exosome standard markers	Biological property could be altered due to alterations in markers on exosomes	[45,56]
PEG isolation	High yield	PEG may affect downstream analysis	[57,58]
